# Protective impact of *Rosa damascena *against neural damage in a rat model of pentylenetetrazole (PTZ)-induced seizure

**Published:** 2020

**Authors:** Mansour Homayoun, Reyhaneh Shafieian, Masoumeh Seghatoleslam, Mahmoud Hosseini, Alireza Ebrahimzadeh-Bideskan

**Affiliations:** 1 *Department of Anatomy and Cell Biology, School of Medicine, Mashhad University of Medical Sciences, Mashhad, Iran.*; 2 *Neurogenic Inflammation Research Center, School of Medicine, Mashhad University of Medical Sciences, Mashhad, Iran.*; 3 *Microanatomy Research Center of Medicinal Plants, School of Medicine, Mashhad University of Medical Sciences, Mashhad, Iran.*

**Keywords:** Rosa damascene, Apoptosis, Seizure, Brain, Neuroscience

## Abstract

**Objective:**

Based on the previously-declared anticonvulsant properties of *Rosa damascena* (*R. damascena*), this study explored the probable effects of *R. damascena* on neuronal apoptosis in the hippocampus of a rat model of pentylenetetrazole (PTZ)-induced seizure.

**Materials and Methods:**

40 male Wistar rats were randomely divided into control (n=8) and experimental (n=32) groups which underwent PTZ injection. A one-week pre-medication with 50 (PTZ-Ext 50) (n=8), 100 (PTZ-Ext 100) (n=8), and 200 (PTZ-Ext 200) (n=8) mg/kg of hydro-alcoholic extract of *R. Damascene *was performed while one experimental group (PTZ-induced group) (n=8) received only saline during the week before PTZ injection. After provocation of PTZ-induced seizures, the brains underwent tissue processing and TUNEL staining assay for apoptotic cell quantification.

**Results:**

Our findings revealed that PTZ-induced seizures led to apoptosis in neuronal cells of all sub-regions of the hippocampus; yet, only at CA1, CA3 and DG sub-regions of the PTZ-induced group, the difference in the number of apoptotic neuronal cells was significant in comparison with the control group. In addition, pre-medication with the plant extract led to a significant drop in the quantity of apoptotic neurons in these sub-regions in comparison with the PTZ-induced group which received no pre-medication .

**Conclusion:**

The results of this study showed that *R. damascena* extract exerts neuro-protective effects on PTZ-induced seizure.

## Introduction

Comprehensive research during previous decades has provided a deep understanding of medicinal plants, especially those with highly beneficial impacts on treatment of human diseases (Gorji, 2003 ▶). *Rosa damascena *(*R. damascena)* is a member of the family* “Rosacea”*, which belongs to the *Rosa *L. genus (Pal, 2013 ▶). Iran, Turkey and Bulgaria, are the main rosarians of the world (Latifi and Minaiyan, 2015 ▶). A great number of organic compounds have been reported to exist in different extracts of *R. damascena, *including citronellol, heneicosane, and disiloxane (Shafei et al., 2011 ▶). This plant is traditionally used to alleviate diverse diseases including painful throats, stomachs, eyes, menstrual cycles, etc. (Libster, 2002 ▶). The hydro-alcoholic extract of *R. damascena *was shown to exert a protective impact on brain tissue oxidative damage (Homayoun et al., 2015 ▶; Mohammadpour et al., 2014 ▶). Moreover, the extract of *R. damascena* has been widely studied and seems to have anti-inflammatory effects (Boskabady et al., 2011 ▶). In addition, anticonvulsant effect of *R. damascena* against pentylenetetrazol (PTZ) or electroshock-induced seizures in experimental animal models has been investigated (Kheirabadi et al., 2008 ▶). Also, the negative effect of *R. damascena* on peroxidation of lipid molecules has been studied (Shafei et al., 2011 ▶). This antioxidant effect might be due to the presence of several important flavonoids in the extract of the plant, such as quercetin 3-O-glucoside (Yassa et al., 2015 ▶). Since oxidative damage has been suggested as a key player in the causation of several central nervous system (CNS) malfunctions, protective impact of *R. damascena *extract versus neural oxidative damage was studied before (Mohammadpour et al., 2014 ▶; Sharma et al., 2009 ▶).

Epilepsy is amongst severe neurological disorders which affects about 50 million people around the world (Goldenberg, 2010 ▶). This condition is mostly characterized by sudden recurrent interruptive neurological attacks, named as epileptic seizures (Seghatoleslam et al., 2015 ▶). Various studies have revealed that epileptic seizures lead to production of destructive free radicals at the cellular level which can damage lipids, proteins and even nuclear DNA (Patel, 2004 ▶). Lipid peroxidation and oxidative injury, as a major result of free radicals production, have been reported to decrease the number of neurons in the cerebral cortex, especially in the hippocampus area (Dam, 1982 ▶). The resulting cell deficiency happening after the seizures, is a consequence of excessive neuron depolarization and extreme glutamate discharge (Stefan and Steinhoff, 2007 ▶).

 According to some evidence, recurrent and single seizures can be induced by a GABA-inhibitor drug like PTZ, which may lead to cell damage or even death in different regions of brain such as hippocampus and the limbic system (Homayoun et al., 2015 ▶). *In vitro* studies have revealed that severe discharges of neurons can lead to cell death, which may appear either as necrosis or apoptosis of granular cells of the dentate gyrus of hippocampus (Meldrum, 2002 ▶). 

Apoptosis is described by the appearance of particular features in the cellular morphology such as cytoplasmic reduction and chromatin condensation, which can be discovered via TUNEL test (Bagheri-abassi et al., 2015 ▶). Some studies have shown that antioxidants may reduce free radicals and subsequently, protect the brain against the damage induced by seizures (Frantseva et al., 2000 ▶).

 Since there is little evidence about medical use of *R. damascena* in reduction of neuronal damage caused by epileptic seizures in animals, we decided to study the impact of hydro-alcoholic extract of this beneficial herb on the apoptosis of hippocampus neurons in a rat model of PTZ-induced seizures.

## Materials and Methods

**Plant extract preparation and related botanical aspects**

*Rosa damascena (Order: Rosales; Family: Rosaceae; Genus: Rosa*) specimens were gathered from Kashan, Iran, and recognized and kept at the Herbarium of School of Pharmacy, Mashhad University of Medical Sciences with the Herbarium No. 254-1804-01. Afterwards, the flower specimens were dried and powdered. The extract was provided using a Soxhlet apparatus by ethanol (70%) and the last product was kept at -4˚C until use. Different concentrations of the final extract were prepared by making a solution of the powder with normal saline, following filtration through a bacterial filter (Abbasnezhad et al., 2015 ▶; Homayoun et al., 2015 ▶; Sarbishegi et al., 2016 ▶). The solution used for injection was prepared freshly every other time. 

**Experimental procedure**

Forty male Wistar rats, aging 8 weeks and weighing 250-300 g, were purchased from and stored at the Animal House of Mashhad University of Medical Sciences, under optimal temperature (22±2°C) and lightning (half-day light vs. dark cycles), with free access to food pellets and water *ad libitum*. All animal procedures were done in full agreement with Mashhad University of Medical Sciences, Ethical Committee Acts. Firstly, the animals were accidentally alienated into ﬁve separate groups (n=8). Three group were considered as the experimental groups since they received a regular day-to-day dose of 50, 100 and 200 mg/kg of *R. damascene *extract (i.p.) for the whole week before being injected with PTZ (100 mg/kg, i.p.) and thus, were named as groups PTZ-Ext 50, PTZ-Ext 100, and PTZ-Ext 200, respectively. The mentioned time period was determined according to a previous study (Pourzaki et al. 2017 ▶). One group served as the control group and received only saline without any PTZ injection. The remaining animals were grouped as PTZ-induced group and received saline during the week before PTZ injection (Rahimi et al., 2018b ▶).

**Tissue sampling**

Two hours after PTZ administration, each rat was sacrificed by ketamine (150 mg/kg, i.p.) and immediately underwent whole-body perfusion through the ascending aorta using 4% formaldehyde. Afterwards, the brains were removed carefully for routine histological processing. Between the points 2.3 to 4.3 mm posterior to the bregma (site of hippocampus), coronal serial sections (8 μm-thickness) were prepared at intervals of every 100 μm. Then, 10 sections from each animal were random selected and carefully mounted on poly-L-lysine-coated slides (Bagheri-abassi et al., 2015 ▶). 

**TUNEL technique**

TUNEL reaction is based upon detection of DNA fragments with the help of terminal deoxynucleotidyl transferase-mediated dUTP nick end-labelling (TUNEL) in nuclei of apoptotic cells using a TUNEL Kit (Roche) (Ataei and Ebrahimzadeh-bideskan, 2014 ▶). After de-parafinization and rehydration, tissue sections were soaked totally in PBS in order to get ready for being treated with 20 μg/ml proteinase K. The activity of cellular endogenous peroxidase was blocked by H_2_O_2_ and after a complete soak in PBS, the sections were incubated overnight in the TUNEL reaction mixture. The day after, all the sections were washed with PBS to be incubated with horseradish peroxidase (POD, 1:500), following treatment with DAB (3,3'Diaminobenzidine) solution in darkness. After constant washing under running water, counterstaining with hematoxylin dye was performed. Through this technique, nuclei of cells affected with apoptosis, can be recognized with kind of dark brown color (Bagheri-abassi et al., 2015 ▶). Positive as well as negative control sections were prepared for assurance of accuracy during the practice, as described before (Ataei and Ebrahimzadeh-bideskan, 2014 ▶).

**Apoptotic cell quantification**

After a careful examination of all sections with 40X objective lens of a light microscope (Bx51/Olympus, Japan), they were photographed using an optical microscope (Olympus, Japan) connected to a computer in order to count TUNEL-positive neuronal cells in all sub-regions of hippocampus including CA1, CA2, CA3 and DG. This activity was completed via using a special counting frame. The mean number of apoptotic neurons per unit area (NA) indifferent sub-regions of hippocampus was counted via the succeeding formula (Bagheri-abassi et al., 2015 ▶):

$\mathrm{NA}=\frac{\sum Q}{\sum P\times a/f}$. 

In this formula, "ΣQ" is the total number of calculated TUNEL-positive cells in each section, while "a/f" is the measured area related to each frame and "ΣP" is the total associated points of each frame which are reaching the reference.

**Statistical analysis**

Statistical analysis in this study was accomplished using SPSS 16 software for windows. Data derived from histological methods were compared using Kruskal-Wallis and Mann-Whitney tests. P**‐**value less than 0.05 was statistically considered significant.

## Results

**The impact of** ***R. damascena*** **extract on the number of apoptotic neurons in the hippocampus**

According to our findings, PTZ-induced seizures induced apoptosis in neuronal cells of all sub-regions of hippocampus; however, significant difference was only observed between the control and PTZ-induced (PTZ) groups at CA1, CA3 and DG sub-regions of the hippocampus. In addition, pre-treatment with 50 (PTZ-Ext 50), 100 (PTZ-Ext 100) and 200 (PTZ-Ext 200) mg/kg of *R. damascena* extract led to a significant reduction in the number of apoptotic neurons in all these sub-regions. 

The mean number of apoptotic neurons per unit area of CA1 sub-region of the hippocampus was 0.33 and 1.40 in control and PTZ-induced groups, respectively; between which, the difference was significant (p<0.01). The mean number of apoptotic neurons per unit area of CA1 sub-region was reduced to 0.70, 0.60 and 0.64 in PTZ-Ext 50, PTZ-Ext 100 and PTZ-Ext 200 groups, in turn. However, only pre-treatment with 100 and 200 mg/kg of *R. damascena* extract led to significant decrease in comparison to PTZ-induced group (p<0.05).

**Figure 1:**
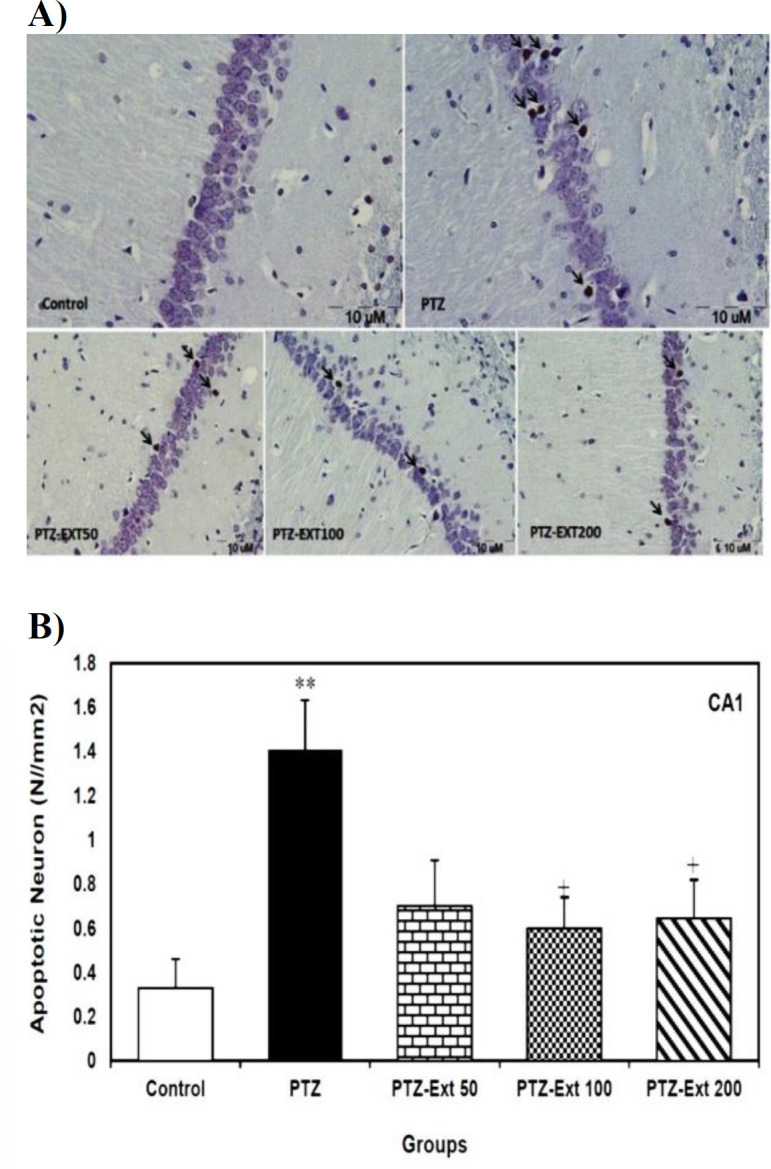
(A) Microscopic images of TUNEL-positive neurons (arrows) in the sections prepared from CA1 sub-region of hippocampus of different groups. TUNEL positive cells, as indicated by shrunk and brown nuclei, are scattered among normal neurons. (B) Comparison of the mean number of apoptotic neurons per unit area of CA1 sub-region in the control, PTZ-induced and pre-treatment groups (n=8)*. *PTZ administration significantly increased the mean number of apoptotic neurons per unit area in comparison to the control group. However, only pre-treatment with 100 and 200 mg/kg of R. damascena extract led to significant decrease in the mean number of apoptotic neurons per unit area in comparison to PTZ-induced group. All data is presented as mean±SEM

 The mean number of apoptotic cells per unit area showed no significant difference among the experimental pre-treatment groups (Figure 1). 

**p<0.01 as compared to the control group; +p<0.05 as compared to the PTZ-induced group.

**Figure 2:**
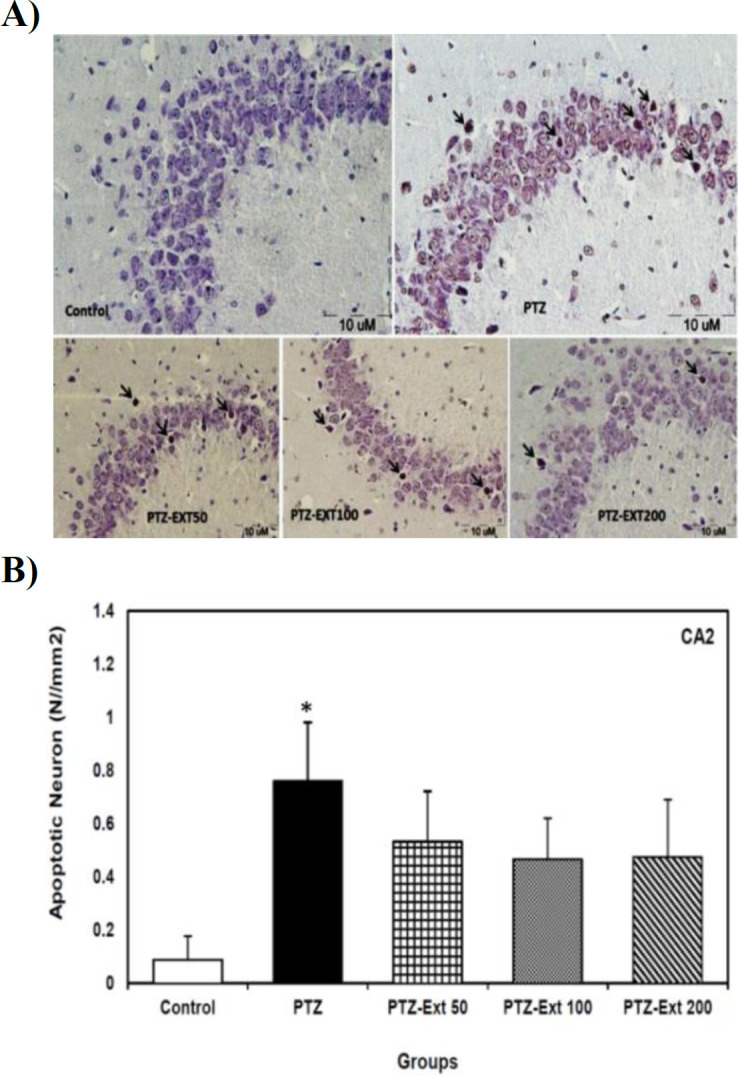
(A) Microscopic images of TUNEL-positive neurons (arrows) in the sections prepared from CA2 sub-region of hippocampus of different groups. TUNEL positive cells are presented with shrunk and brown nuclei, dispersed among normal neurons. (B) Comparison of the mean number of apoptotic neurons per unit area of CA2 sub-region in the control, PTZ-induced and pre-treatment groups (n=8)*.* Although PTZ administration significantly increased the mean number of apoptotic neurons per unit area in comparison to the control group, no significant decrease was observed in pre-treatment groups when compared to PTZ-induced group. All data is presented as mean±SEM

The mean number of apoptotic neurons per unit area of CA2 sub-region was 0.08 and 0.76 in the control and PTZ-induced group, respectively; which was reduced to 0.53, 0.46 and 0.47 in PTZ-Ext 50, PTZ-Ext 100 and PTZ-Ext 200 groups, in turn. Although PTZ administration significantly increased the mean number of apoptotic neurons per unit area in comparison to the control group (p<0.05), no significant decrease was shown in pre-treatment groups when compared to the PTZ-induced group. Additionally, the mean number of apoptotic cells per unit area exhibited no significant difference among the experimental groups (Figure 2). 

*p<0.05 compared to the control group.

**Figure 3:**
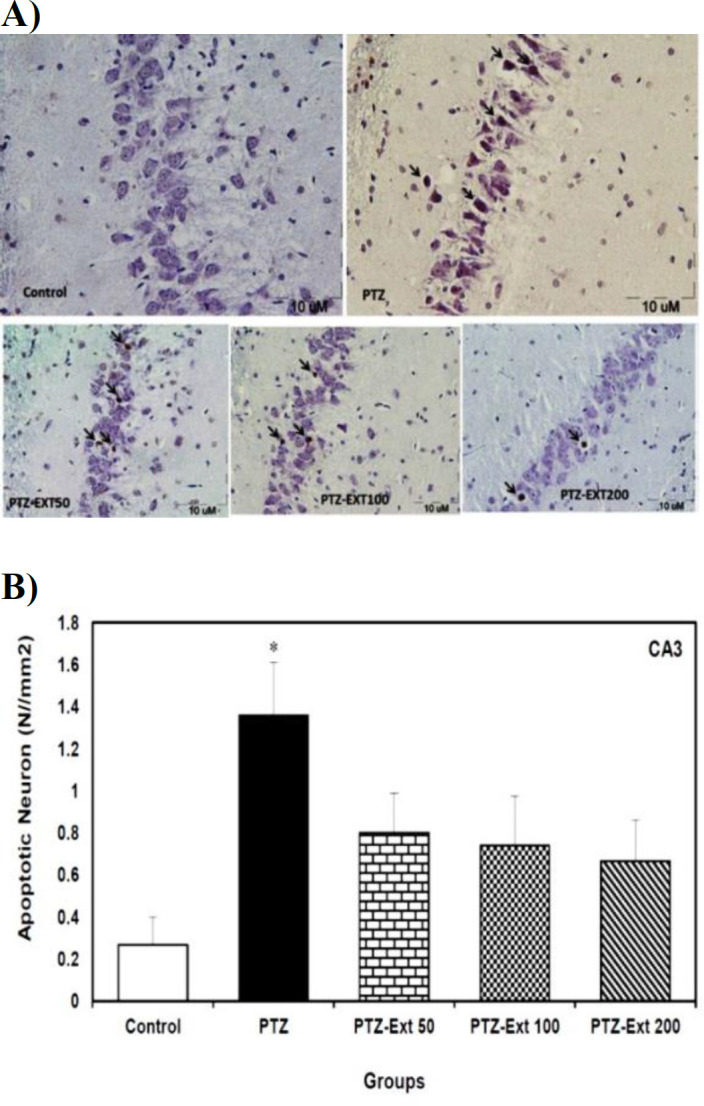
(A) Microscopic images of TUNEL-positive neurons (arrows) in the sections prepared from CA3 sub-region of hippocampus of different groups. TUNEL positive cells, revealed by shrunk and brown nuclei, are spread among normal neurons. (B) Comparison of the mean number of apoptotic neurons per unit area of CA3 sub-region in the control, PTZ-induced and pre-treatment groups (n=8)*. *Although PTZ administration significantly increased the mean number of apoptotic neurons per unit area in comparison to the control group, no significant decrease was found when comparing the pre-treatment groups with PTZ-induced group. All data is presented as mean±SEM

The mean number of apoptotic neurons per unit area of CA3 sub-region was 0.26 and 1.36 in the control and PTZ-induced groups, respectively; which was reduced to 0.80, 0.74 and 0.66 in PTZ-Ext 50, PTZ-Ext 100 and PTZ-Ext 200 groups, in turn. Although the alteration was significant between the control and PTZ-induced group (p<0.05), no significant difference was observed when comparing pre-treatment groups with the PTZ-induced group. Furthermore, the mean number of apoptotic cells per unit area revealed no significant difference among the experimental pre-treatment groups (Figure 3).

*p<0.05 compared to the control group.

**Figure 4:**
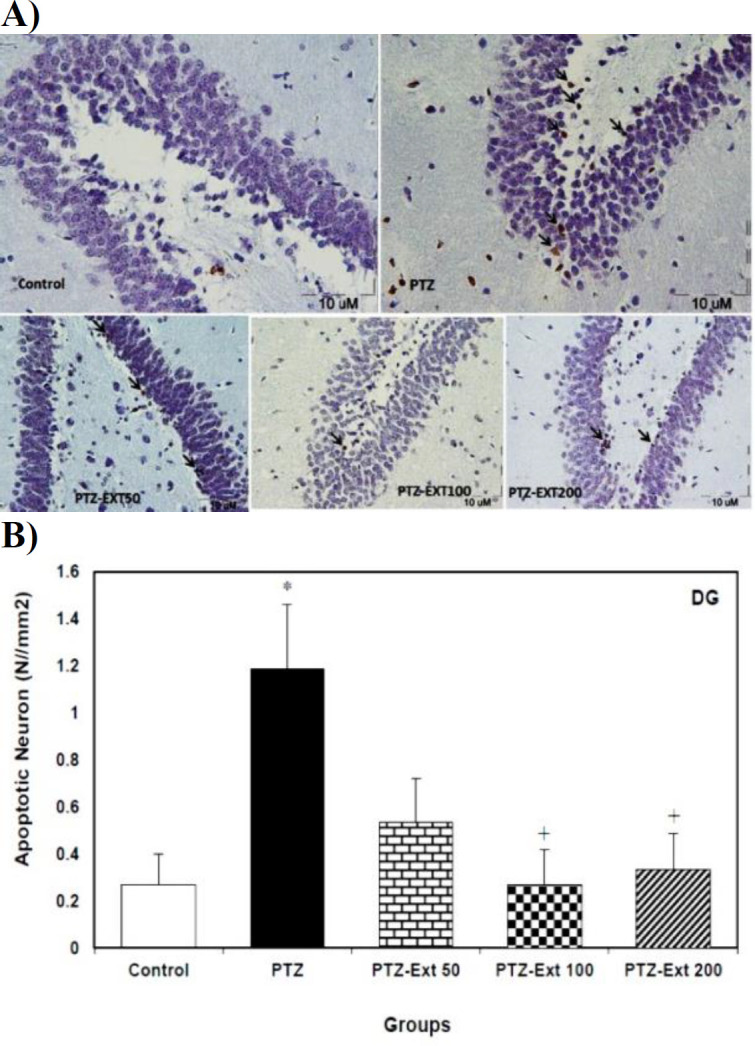
(A) Microscopic images of TUNEL-positive neurons (arrows) in the sections prepared from DG sub-region of hippocampus in different groups. TUNEL positive cells, with shrunk and brown nuclei, are scattered among normal neurons. (B) Comparison of the mean number of apoptotic neurons per unit area of DG sub-region in the control, PTZ –induced and pre-treated groups (n=8)*. *PTZ administration significantly increased the mean number of apoptotic neurons per unit area in comparison with the control group. However, only pre-treatment with 100 and 200 mg/kg of R. damascena extract led to significant decrease in the mean number of apoptotic neurons per unit area in comparison to PTZ-induced group. All data is presented as mean±SEM

The mean number of apoptotic neurons per unit area of DG sub-region was 0.26 and 1.18 in the control and PTZ-induced groups, respectively; which were significantly different (p<0.05). The mean number of apoptotic neurons per unit area of DG sub-region was decreased to 0.53, 0.26 and 0.33 in PTZ-Ext 50, PTZ-Ext 100 and PTZ-Ext 200 groups, in turn. However, only pre-treatment with 100 and 200 mg/kg of *R. damascena* extract led to significant decrease (p<0.05) in comparison to PTZ-induced group. The mean number of apoptotic neurons per unit area showed no significant difference among the pre-treatment groups (Figure 4).

*p<0.05 compared to control group; +p<0.01 compared to PTZ-induced group.

## Discussion

Previously, we reported that hydro-alcoholic form of *R. damascena *extract could significantly reduce the frequency and severity of epileptic seizures in a PTZ-induced model of epilepsy in rats (Homayoun et al., 2015 ▶). We also reported that the extract could significantly decrease the number of dark neurons appeared in the hippocampus of an epileptic animal model (Homayoun et al., 2015 ▶). In this study, the neuro-protective effect of hydro-alcoholic extract of *R. damascena *on occurrence of apoptosis in hippocampus neurons was investigated. 

According to our findings, PTZ-induced seizures induced apoptosis in neuronal cells of all sub-regions of hippocampus; however, significant differences were only observed between the control and PTZ-induced groups at CA1, CA3 and DG sub-regions of hippocampus. In addition, pre-treatment with *R. damascena* extract led to a significant reduction in the number of apoptotic neurons in all these sub-regions. 

WHO has reported that about 50 million people are dealing with epilepsy worldwide; of which, near 80% inhabit in low and middle-income countries (Emilie et al., 2019 ▶), where herbal medications are usually known as the common form of complementary medicine used for seizure control (Liu et al., 2017 ▶). Moreover, epilepsy is one of the most challenging neurological disorders that almost always requires a lifetime-long period of medical treatment with anti-epileptic drugs (AEDs), which have well-known side-effects such as sedation, hepatotoxicity, anxiety and depression (Gohil and Enhoffer, 2014 ▶; Vossler et al., 2018 ▶). Nevertheless, application of herbal medications ought to be subjected to more detailed evidence-based researches to clarify the exact mechanisms and effects. Two of the most-studied anticonvulsant herbs, especially in Iran, are *Nigella sativa* (black cumin) and *Cannabis sativa* (cannabis) (Zimmerman and Yarnell, 2018 ▶). Thus, we decided to investigate the possible neuro-protective effects of *R. damascena* on hippocampus neurons apoptosis following seizure induction by PTZ. 

Seizures were revealed to induce oxidative stress and inflammation, both of which lead to extensive neuronal cell loss (Vezzani et al., 2013 ▶). Indeed, not only production of reactive oxygen species (ROS) due to seizures causes neuronal cell loss, but also release of pro-inflammatory cytokines induces cell injury; mostly in form of apoptosis (Shin et al., 2011 ▶; Teocchi and D’Souza-Li, 2016 ▶). It has been well documented that seizures lasting for more than 30 min can be the main reason for apoptosis in both experimental animals and humans (Bengzon et al., 2002 ▶). Thus, it seems that administration of a kind of medication, with anti-inflammatory and antioxidant features, may be a wise approach to exert beneficial effects against neuronal cell loss in the brain.

*R. damascena* is a popular aromatic plant that is renowned for its fragrance and flavorings as well as anti-depressant and anti-anxiety effects (Mohebitabar et al., 2017 ▶; Pal, 2013 ▶). Studies have revealed that beside its common use in traditional medicine as a hypnotic and antiseptic agent, extract of *R.* *damascena* possesses a broad spectrum of medical effects, including antimicrobial, antidepressant, anti-inflammatory and antioxidant activity (Libster, 2002 ▶). As oxidative stress is one of the major etiologies suggested for complications resulting from epileptic seizures (Aguiar et al., 2012 ▶; Shin et al., 2011 ▶), we assumed that extract of *R. damascena* may have a protective effect against oxidative damage in the hippocampus. The results of this study showed that application of 100 and 200 mg/kg of hydro-alcoholic extract of *R. damascena* led to a significant decrease in the number of apoptotic neurons in CA1 and DG sub-regions of hippocampus.

The antioxidant effect of *R. damascene* has been attributed to the great amount of phenolic compounds, responsible for its bitter taste, which exerts a major inhibitory effect on lipid peroxidation and oxidative stress (Baydar and Baydar, 2013 ▶; Kumar et al., 2009 ▶; Memariani et al., 2015 ▶). In addition, quercetin-3-O-glucoside, one of the chief flavonoids present in the extract of this plant, has been shown to have antioxidant and inflammatory activity (Yassa et al., 2015 ▶). A neuro-protective effect of *R. damascene,* due to its antioxidant compounds, has also been claimed in another study (Mohammadpour et al., 2015 ▶). As a whole, this mechanism could be considered as one of the main explanations for the protection of brain tissue against neuronal apoptosis following induction of seizures. However, the exact molecular mechanisms surely need to be clarified in the forthcoming studies.

Different dosages of the extract of *R. damascena* have been studied before. Most of the previous studies used essential oil of this plant to investigate the possible therapeutic effects (Babaie et al., 2007 ▶; Keyhanmehr et al., 2018 ▶; Kheirabadi et al., 2008 ▶; Maleki et al., 2013 ▶; Ramezani et al., 2008 ▶). However, we decided to evaluate the hydro-alcoholic extract of *R. damascene *in this study as it has been investigated little before (Rezvani-Kamran et al., 2017 ▶). Indeed, most of these studies focused on medium to high dosages of the hydro-alcoholic extract of this plant e.g. 250, 500 and 1000 mg/kg (Baniasad et al., 2015 ▶; Hajhashemi et al., 2010 ▶; Rahimi et al., 2018a ▶; Shafei et al., 2015 ▶). Thus, a lower range of different dosages of the hydro-alcoholic extract of *R. damascene *was employed in this study (i.e. 50, 100 and 200 mg/kg) in order to find the lowest optimal dose of the herb that could also be beneficial.

PTZ is a tetrazol derivative that was first reported by Mason and Cooper (1972) ▶ in a rat model and used to be usually prescribed as a medical drug against depression (Mason and Cooper, 1972 ▶). At the cellular level, PTZ acts as an agonist to the ion channels which primarily transport sodium and calcium. More-ever, it affects the interactions with the gamma aminobutyric acid (GABA) receptors. Thus, high doses of PTZ can induce burst discharges in neuronal cells that appear as tonic-clonic seizures (Jefferys, 2003 ▶). In line with our previous study, other studies confirmed that PTZ-induced seizure can damage neuronal structure of hippocampus, which is one of the most sensitive regions of the brain (Homayoun et al., 2015 ▶; Seghatoleslam et al., 2015 ▶). Although PTZ-induced seizures cause apoptosis in neuronal cells of all sub-regions of hippocampus in this study, significant difference in the mean number of apoptotic neurons between the control and PTZ-induced groups was only recognizable in CA1, CA3 and DG sub-regions of the hippocampus; of which, only the mean number of apoptotic neurons in CA1 and DG sub-regions was significantly reduced after pre-treatment with 100 and 200 mg/kg of hydro-alcoholic extract of *R. damascene*. Indeed, these two doses revealed no significant protective effect against PTZ-induced neuronal apoptosis in CA3 sub-region of hippocampus. It was proposed that CA3 pyramidal neurons may show different responses to external modifications due to their specific intrinsic features (Avoli, 2007 ▶). However, it seems that a more clear explanation should be given by future studies. Besides, the exact compounds responsible for the neuro-protective effect of *R. damascene* are not investigated in the present study and this issue should also be explored in the future. Although thousands of studies have claimed that herbal medications may be beneficial to epilepsy, most of them are at experimental levels and performed in animals; thus, due to lack of data on their possible efficacy as well as adverse effects in clinical use, more trials are still needed. Thus, it seems so necessary to put much more emphasis on this issue. Another weak point of this study was the limited molecular survey on the exact pathway of apoptosis being prevented by *R. damascene*, which must be investigated in the time ahead. 

The results of the present study revealed, for the first time, that the hydro-alcoholic extract of *R. demascena* exerts potential neuro-protective effects in a rat model of seizure through significant reduction of apoptotic neurons in several sub-regions of hippocampal formation.
